# Predicting the acute aquatic toxicity of organic UV filters used in cosmetic formulations

**DOI:** 10.5599/admet.2364

**Published:** 2024-09-11

**Authors:** Chrysanthos Stergiopoulos, Fotios Tsopelas, Maria Ochsenkühn-Petropoulou, Klara Valko

**Affiliations:** 1 Laboratory of Inorganic and Analytical Chemistry, School of Chemical Engineering, National Technical University of Athens, Iroon Polytechneiou 9, Zografou 157 80, Athens, Greece; 2 Business & Technology Centre, Bessemer Drive, Stevenage, Herts, SG1 2DX, United Kingdom

**Keywords:** UV filters, aquatic toxicity, phospholipid binding, IAM chromatography, octanol-water partition coefficient, environmental risk assessment

## Abstract

**Background and purpose:**

Organic UV filters are commonly used in sunscreen and cosmetic formulations to protect against harmful UV radiation. However, concerns have emerged over their potential toxic effects on aquatic organisms. This study aims to investigate the acute aquatic toxicity of 13 organic UV filters and determine whether phospholipid binding, measured through biomimetic chromatographic methods, is a better predictor of toxicity than the traditionally used octanol-water partition coefficient (log *P*).

**Experimental approach:**

The chromatographic retention of the 13 UV filters was measured on an immobilized artificial membrane (IAM) stationary phase to assess phospholipid binding. These measurements were then applied to previously established predictive models, originally developed for pharmaceutical compounds, to estimate acute aquatic toxicity endpoints of 48-hour LC_50_ for fish and the 48-hour EC_50_ (immobilization) for Daphnia magna.

**Key results:**

Phospholipid binding was found to be a more reliable predictor of the acute aquatic toxicity of UV filters compared to log *P*. The toxicity was primarily driven by lipophilicity and charge, with negatively charged compounds exhibiting lower toxicity.

**Conclusion:**

The study demonstrates that phospholipid binding is a better descriptor of UV filter toxicity than log *P*, providing a more accurate method for predicting the environmental risk of these compounds. This insight can guide the development of more environmentally friendly sunscreens by reducing the use of highly lipophilic and positively charged compounds, thus lowering their aquatic toxicity.

## Introduction

Sun exposure is one of the primary contributors to extrinsic skin aging, profoundly impacting the skin. It is estimated to cause up to 90 % of visible skin aging, especially in individuals lacking the natural protection provided by higher levels of melanocytes [1]. Photoaged skin becomes wrinkled, lax, rough, and unevenly pigmented, with increased epidermal thickness and connective tissue alterations [2]. Thus, protecting the skin from prolonged sun exposure is crucial to reduce photoaging. Mineral sunblocks, such as titanium dioxide, zinc oxide, and various organic compounds, can be applied topically to shield the skin from harmful UVA and UVB rays. By 2005, over 300 products marketed as sun protection were available [3], containing more than 25 different UV filter chemicals. Most sun protection products function by absorbing, reflecting, or scattering sunlight [4]. In the USA, chemical UV filters must be approved by the FDA and are utilized in various sunscreen and cosmetic products [5].

**Figure 1.**
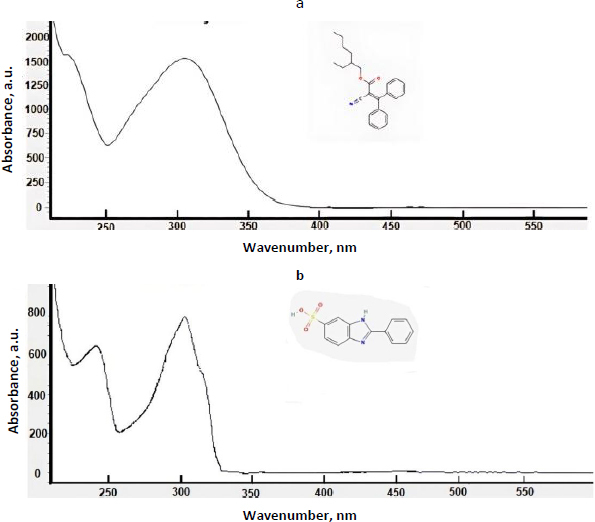
UV spectra of two investigated UV filter compounds used in sunscreen products: UV spectrum of a) octocrylene and b) ensulizole

The effectiveness of many organic UV filters is based on their ability to absorb UV radiation. Their effectiveness is measured by the Sun Protection Factor (SPF) [6]. Most sunscreen products contain active ingredients that protect against UVB rays, which have wavelengths ranging from 290 to 320 nm and are the primary cause of sunburn. Some sunscreens also protect against UVA rays, which range from 320 to 400 nm, penetrate more deeply into the skin and can cause cancer [7]. In the USA, the FDA considers these compounds as drugs that must be approved for cosmetic formulations, while European law classifies them as cosmetic ingredients [8]. These compounds typically contain aromatic rings with various substituents and exhibit UV absorbance spectra with two maxima, one above 320 nm and the other below 300 nm [7]. In this study, we investigated the properties of thirteen synthetic organic compounds used in sunscreen formulations. Figure 1 shows typical UV spectra with the characteristic double maxima of UV absorbance for two UV filter compounds.

Sunscreen products can enter the marine environment both directly through activities like swimming and bathing and indirectly through wastewater discharges. Wastewater treatment plants are often unable to efficiently remove high concentrations of organic UV filters [9], and the natural degradation of these compounds is slow, leading to their accumulation in the environment. These compounds are present in effluents and freshwater sources that eventually reach the sea. The first analyses of organic UV filters in swimming pools and seawater samples were conducted in 2002 [9]. Since 2015, there has been an increase in publications investigating the effects of sunscreen products on coral reefs and various coastal areas [10,11]. The impact depends on exposure levels, emissions, and resulting field concentrations.

In coastal areas, the release of UV filter compounds into natural waters is inevitable due to the large amounts of personal care products produced and used. These formulations are continuously introduced into the aquatic environment during regular use, primarily through municipal sewage treatment plants [12]. The occurrence and concentrations of the seven most frequently used UV filters in river and lake sediments were investigated over six months using gas chromatography-mass spectrometry [13]. UV filter concentrations in river sediments remained low and constant over time, while lake sediments showed high levels during summer, with concentrations dropping in autumn. Cosmetic ingredients can also be found in human wastewater, eventually entering the environment unless specifically removed from the wastewater system [14,15]. In this way, sunscreen compounds contaminate the environment similarly to pharmaceutical compounds taken orally.

Various studies have documented the adverse effects of UV filters on marine environments and organisms, including mortality, growth inhibition, reproduction failure due to endocrine disruption, coral bleaching, and accumulation in food chains [16,17]. Tsui *et al.* [18] analysed UV filter occurrences, distribution, and potential ecological risks in various coastal areas, identifying the most contaminated regions as Hong Kong, Spain, Los Angeles, New York, and Oslo. A 2015 review highlighted sunscreen compounds as a new environmental risk associated with coastal tourism, listing common sun protection compounds, their concentrations in various products, and their presence in coastal waters and rivers [19]. Recent studies have revisited the toxic effects of UV filters from sunscreens on coral reefs, suggesting they may contribute to coral decline at an unprecedented pace. Regulatory measures should be thoroughly evaluated based on actual evidence [11]. The complexity of sunscreen products makes it challenging to replace one UV filter with another, as multiple compounds are usually required to achieve sufficient broad-spectrum protection.

**Figure 2.**
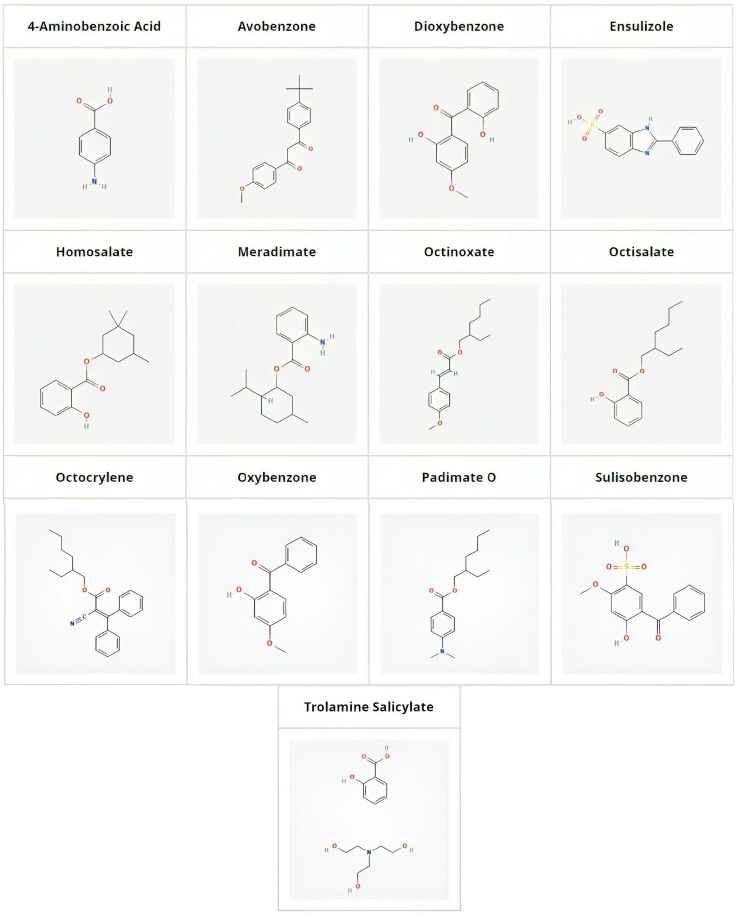
Structure of investigated UV filter compounds

UV filter compounds are found in high concentrations in coastal areas frequented by tourists, posing a significant risk to aquatic life. Therefore, evaluating the aquatic toxicity of these compounds is crucial for the cosmetic industry to select chemicals that are least harmful to marine environments. In our previous publications, the aquatic toxicity of pharmaceuticals has been modelled using various measured and calculated properties of compounds [20]. It was discovered that biomimetic HPLC measurements, particularly phospholipid binding [21,22], can predict the aquatic toxicity of xenobiotics. Biomimetic HPLC measurements using an immobilized artificial membrane (IAM) stationary phase offer a fast and reliable method to measure the phospholipid binding of compounds, which correlates with their toxicity [23]. In this study, the phospholipid binding of thirteen compounds used in various sunscreen products is measured and their aquatic toxicity is predicted using established model equations [20]. The UV filters investigated, along with their structures, are shown in Figure 2. It is highlighted that the UV filter effect is not related to lipophilicity and, consequently, not to the aquatic toxicity of the compounds. This conclusion can assist in selecting UV filters that pose the least potential risk to the environment and aquatic life in natural waters.

## Experimental

### UV filters under investigation

A total of thirteen UV filter compounds commonly used in cosmetic formulations were investigated in this manuscript and were obtained from Sigma-Aldrich (Merck, Gillingham, Dorset, UK). These include, in alphabetical order, the following compounds: 4-aminobenzoic acid, avobenzone, dioxybenzone, ensulizole, homosalate, meradimate, octinoxate, octisalate, octocrylene, oxybenzone, padimate O, sulisobenzone and trolamine salicylate. The compounds were dissolved in dimethylsulfoxide at 10 mmol/L concentration and 10 μL stock solutions were diluted down with DMSO to 100 μL before injecting 5 μl into an Agilent 1100 HPLC system. Chromatograms were recorded using a photodiode array detector (DAD) monitoring 220, 230, 254 and 280 nm.

### Database search for experimental ecotoxicological values

Experimental 48 h EC_50_ values (immobilization) to water flea (*Daphnia magna*) and 96 h LC_50_ values to fish species expressed in mg/L were collected from the European Chemical Agency database (ECHA) [24] and converted into molar concentrations (M) before being converted to their negative logarithm p values (-log). All values are presented in Table 1S in the Supplementary material.

### Physicochemical properties

ADME Boxes v.3.0 software (Advanced Chemistry Development Inc., Toronto, ON, Canada) was used to extract various physicochemical parameters of the investigated compounds, such as octanol-water partition (log *P*) and distribution at pH value of 7.4 (log *D*_7.4_) coefficients, hydrogen bond donor (HBD) and acceptor (HBA) groups, Abraham’s hydrogen bond acidity (*A*) and basicity (*B*), total polar surface area (TPSA), molecular weight (MW), as well as the molecular fractions of positively charged (*F*^+^), negatively charged (*F*^-^) and zwitterionic (*F* ^z^) species at pH 7.4. The collected physicochemical parameters are presented in Table 1.

**Table 1.** Physicochemical properties of UV-filter compounds investigated, values of *F*^+^ and *F* ^z^ are 0.00 for all compounds

Compound	log *P*	log *D*_7.4_	MW, g/mol	TPSA, Å^2^	*F* ^-^	*A*	*B*	HBD	HBA
4-Aminobenzoic Acid	0.83	-1.60	137	63.2	1.00	0.94	0.60	3.00	3.00
Avobenzone	4.58	4.53	310	43.4	0.10	0.00	1.08	0.00	3.00
Dioxybenzone	2.99	2.89	244	66.8	0.18	0.41	0.70	2.00	4.00
Ensulizole	0.31	-3.73	274	91.4	1.00	0.66	1.43	2.00	5.00
Homosalate	4.90	4.90	262	46.5	0.00	0.13	0.49	1.00	3.00
Meradimate	4.64	4.64	275	52.3	0.00	0.18	0.83	2.00	3.00
Octinoxate	5.71	5.71	290	35.5	0.00	0.00	0.78	0.00	3.00
Octisalate	5.10	5.10	250	46.5	0.00	0.13	0.45	1.00	3.00
Octocrylene	7.27	7.27	361	50.1	0.00	0.00	0.86	0.00	3.00
Oxybenzone	3.50	2.89	228	46.5	0.18	0.13	0.62	1.00	3.00
Padimate O	5.31	5.31	277	29.5	0.00	0.00	0.84	0.00	3.00
Salicylic acid (Trolamine Salicylate)	2.04	-1.89	138	57.3	1.00	0.71	0.38	2.00	3.00
Sulisobenzone	0.28	-4.71	308	109	1.00	0.45	1.37	2.00	6.00

### Predictive ecotoxicity models

Models based on the phospholipid binding CHI_IAM_ of a set of pharmaceutical compounds for the prediction of their acute aquatic toxicity to fish species (48 h LC_50_) and water flea (*Daphnia magna*) (48 h EC_50_) established in our previous publication [20] were used in order to make new predictions regarding the acute aquatic toxicity of the UV filter compounds. Corresponding models constructed based on log *P* values were also used for comparison. The models, Equations (1) to (4), along with their statistical parameters (determination coefficients - *R*, *R*^2^ and adjusted *R*^2^, *R*^2^_adj_, standard deviation - *s* and Fisher test - *F*) are presented in Table 2. In addition, Tables 2S and 3S in the Supplementary material include the training set of the pharmaceutical compounds used for the model construction, their CHI_IAM_ values, and their physicochemical parameters, respectively.

**Table 2.** Predictive models used for UV-filter ecotoxicity predictions

Organism	Equation	*R*	*R* ^2^	*R* ^2^_adj_	*s*	*F*
Fish	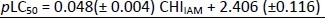 (1)	0.924	0.854	0.848	0.384	157
	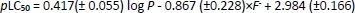 (2)	0.858	0.735	0.715	0.527	36.1
Water flea (*Daphnia magna*)	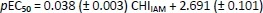 (3)	0.907	0.823	0.817	0.335	125
	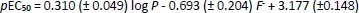 (4)	0.816	0.665	0.639	0.471	25.8

### Measurement of phospholipid binding using IAM chromatography

The phospholipid binding was measured using an IAM PC.DD2 column with dimensions of 100×4.6 mm (Regis Technologies Inc., Morton Grove, IL, USA). The gradient retention times were measured using a 50 mmol/L ammonium acetate mobile phase with the pH adjusted to 7.4. The mobile phase flow rate was 1.5 mL/min. The acetonitrile gradient was applied to reach 90 % in 4.75 min. The 90 % acetonitrile concentration was maintained for an additional 0.5 min (to 5.25 min) and returned to 0 % by 5.5 min. The cycle time was 6 min, plus an additional 1 min equilibration time was applied while the injector prepared for the next injection. The standard deviation in the retention time measurements was ±0.005 min derived from 3 repeated injections. The gradient retention times were calibrated with the acetophenone homologues for which the Chromatographic Hydrophobicity Index values on the IAM column (CHI_IAM_) have been established using isocratic measurement [25]. The standard error ranged from 0.1 to 0.8 CHI_IAM_ values. CHI_IAM_ approximates the acetonitrile concentration in the mobile phase when the compound elutes. Table 4S in the Supplementary material shows the calibration set of compounds and their predetermined CHI_IAM_ values. CHI_IAM_ values above 45 indicate strong phospholipid binding.

### Ecotoxicity predictions of EPI Suite software

EPI Suite Software v.4.11 (US EPA, Washington, DC, USA) was used to obtain predicted ecotoxicological endpoints since this software is widely accepted in environmental sciences [26]. The ECOSAR module implemented in EPI Suite software was employed to predict 96 h LC_50_ values for fish and 48 h LC_50_ values for daphnids. They are presented in Table 5S in the Supplementary material. It should be noted that for a single given structure, the ECOSAR module may provide more than one result if the entered molecule contains the base structure from multiple classes as identified in the ECOSAR class definition sheets. In such cases, the value corresponding to the most suitable class of the UV filter under study was considered.

### Statistical software and methods

JMP v.13.0 (SAS Institute Inc., Cary, NC, USA) and SPSS 23.0 (IBM SPSS Statistics, Chicago, IL, USA) were used for the statistical calculations and the principal component analysis (PCA).

The predictive ability of the models was evaluated using residual values (*e*_i_), root mean square error of prediction (RMSEP), relative standard error of prediction (RSEP, %) and bias. Residual values were defined as:



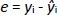

(5)


where *y*_i_ is the observed (experimental) value of sample i and *ŷ*_i_ the predicted value of sample i. In that aspect, RMSEP was calculated according to Equation 6:



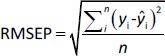

(6)


where *n* is the total number of test compounds. Subsequently, RSEP and bias were calculated using Equations (7) and (8), respectively:



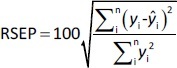

(7)




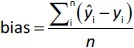

(8)


Applicability domain of the models was defined using Williams Plot by plotting (externally) studentized residuals versus leverage values. Leverage *h*_i_ is defined by Equation (9):



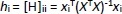

(9)


where *x*_**i**_ is the descriptor row-vector of the query compound, and *X* is the *n*×*p* matrix of *p* model descriptor values for *n* training set compounds. The superscript T refers to the transpose of the matrix/vector. The warning leverage h* was fixed at (Equation (10)):



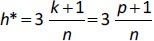

(10)


where *n* is the number of training compounds and *k* is the number of model parameters plus one (*p* + 1).

Studentized residuals are equal to:



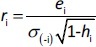

(11)


where *σ*_(-i)_ is the standard error of the residuals excluding the i^th^ observation.

## Results and discussion

### Investigating the physicochemical profile of UV filters

**Figure 3.**
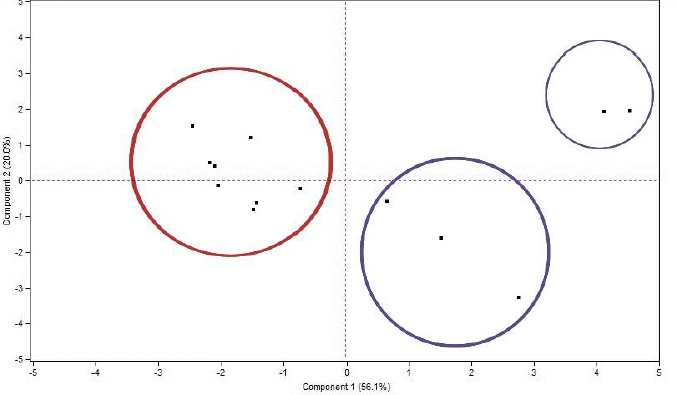
Score plot of the investigated UV filters based on their physicochemical properties

Before predicting the ecotoxicity of UV filter compounds, their physicochemical profile was scrutinized by performing a PCA score plot based on their physicochemical properties presented in Table 1. During the PCA, six principal components were extracted with cumulative determination coefficient *R*^2^_cum_ = 0.911 and cross-validated correlation coefficient *Q*^2^_cum_ = 0.539. The scores of the compounds were plotted between the first two components, which explains 76.1 % of the variance, as seen in Figure 3.

Based on the position of the compounds in the plane between the two components, it is evident that there is a considerable variance in the properties among the investigated compounds. One primary group can be identified with respect to the position of the compounds with the first component. More specifically, this group is found on the lefthand of the first component and includes the compounds avobenzone, homosalate, meradimate, octinoxate, octisalate, octocrylene, oxybenzone and padimate O. It is labelled in Figure 3 with a red circle. The main characteristics of the aforementioned compounds are their high lipophilicity values (log *P* > 4.5) and their predominantly non-ionizable character. Taking into account that high lipophilicity values, especially log *P* greater than 5, show an increased risk for promiscuity and toxicity [27], it is important to investigate the toxic potential of such compounds to aquatic organisms.

All other compounds fall on the righthand side of the first component and are far away from the first group. These compounds include the following: aminobenzoic acid, dioxybenzone, ensulizole, sulisobenzone and trolamine salicylate and are labelled in Figure 3 with blue circles. Contrary to the previous group, these compounds are much less lipophilic (log *P* < 3) and negatively charged at pH 7.4. This difference in their physicochemical properties in comparison to the first compound group explains their distinguished positioning on the plane. These compounds can be further divided into two subgroups according to the second component, with the compounds ensulizole and sulisobenzone lying above the second component and further away from the rest of the compounds, while the compounds aminobenzoic acid, dioxybenzone and trolamine salicylate being located below the second component.

### Biomimetic HPLC phospholipid binding

Table 3 contains the HPLC phospholipid binding data (CHI_IAM_) of the thirteen UV filter molecules investigated. As seen from Table 3, the investigated compounds show mainly either strong or weak phospholipid binding.

**Table 3.** Chromatographic phospholipid binding of investigated UV filters

Compound	CHI_IAM_
4-Aminobenzoic Acid	3.30
Avobenzone	53.4
Dioxybenzone	39.9
Ensulizole	13.2
Homosalate	56.8
Meradimate	56.9
Octinoxate	54.2
Octisalate	55.1
Octocrylene	60.0
Oxybenzone	40.8
Padimate O	53.6
Sulisobenzone	14.3
Trolamine salicylate (Salicylic acid)	1.13

Strong phospholipid binding (CHI_IAM_ > 45) can be attributed to high lipophilicity, since hydrophobicity is the predominant force of partitioning [28]. Indeed, compounds that show such CHI_IAM_ values, such as avobenzone, homosalate, meradimate, octinoxate, octisalate and octocrylene, possess at the same time higher log *P* values (> 4.5). This characteristic is further enhanced by their lack of ionization. On the other hand, compounds with weaker phospholipid binding (CHI_IAM_ < 20), such as aminobenzoic acid, sulisobenzone, ensulizole and trolamine salicylate, are less lipophilic (log *P* < 2) and 100 % negatively charged at pH 7.4. It also means that they form a salt with the mobile phase buffer. Trolamine has no UV, and it is not detected in our measurements. Only salicylic acid plays a role in the sunscreen effect. Trolamine salt reduces skin irritation caused by salicylic acid in sunscreen products. Therefore, the trolamine salicylate salt was handled as salicylic acid. Taking into account that the negative charge of phosphate anions of phospholipids is located on the surface of the membrane, the negative charge of the compounds contributes even more to the decrease of their phospholipid binding [29]. An intermediate behavior is shown by dioxybenzone, which, along with a log *P* value of 3 and an 18 % negatively charged molar fraction at pH of 7.4, exhibits a CHI_IAM_ value of 39.9.

The immobilized artificial membrane (IAM) stationary phase has been designed to mimic a lipid membrane on a solid surface. This phase features phosphatidylcholine chemically bonded to a silica stationary phase. Chromatographic retention on the IAM phase is proportional to a compound’s distribution between the mobile phase (at physiological pH) and the phospholipid surface of the stationary phase. This retention mechanism differs from the octanol/water partitioning commonly used to assess lipophilicity and environmental toxicity of compounds. Research has shown that IAM lipophilicity closely matches octanol/water lipophilicity for nonpolar, neutral compounds, according to the Abraham solvation equation approach [25,30]. However, the behaviour of charged compounds differs: while octanol/water log *D* values decrease for both positively and negatively charged compounds, IAM retention decreases for negatively charged compounds but increases for positively charged ones [31-34]. Additionally, steric effects influence IAM retention, with elongated molecules exhibiting stronger retention compared to round-shaped molecules. Given that compounds partitioning into phospholipid membranes may disrupt cellular membranes, IAM retention could be a better predictor of toxicity than octanol/water partition coefficients.

### Predicting the acute aquatic toxicity of UV filters

#### Applicability domain of predictive models

**Figure 4.**
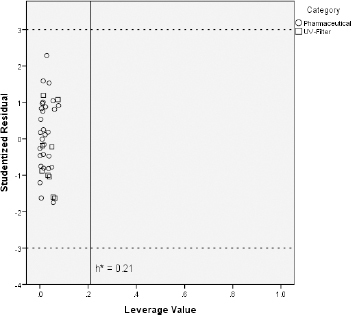
Applicability domain of fish pLC_50_ CHI_IAM_ predictive model

**Figure 5.**
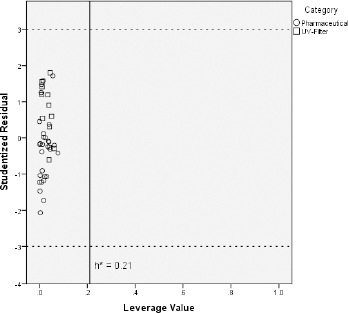
Applicability domain of *daphnia* pEC_50_ CHI_IAM_ predictive model

Some investigated UV filters exhibit slightly higher CHI_IAM_ values than the pharmaceuticals on which the predictive models were trained. For instance, octocrylene, homosalate and meradimate possess CHI_IAM_ values of 60.0, 56.8 and 56.9, respectively, when the highest CHI_IAM_ value of the pharmaceuticals was 55.3 for the drug amitriptyline. Additionally, CHI_IAM_ values of avobenzone, octinoxate, octisalate, and padimate O belong close to this upper limit of pharmaceutical phospholipid binding. For that reason and in order to ensure that the previously established models can be used for reliable predictions regarding the toxicity of UV filters, the applicability domain of the CHI_IAM_ models was defined with the use of the Williams plot [35,36]. The aforementioned plots were constructed by plotting the leverage values of each compound (*h*_i_) vs the corresponding studentized residuals (*r*_i_). The plots are illustrated in Figure 4 in the case of fish and Figure 5 in the case of *Daphnia* toxicity.

Figures 4 and 5 show that all the UV filter compounds fall into the applicability domain of CHI_IAM_ models since all compounds possess leverage values below the critical leverage (h*), as defined by Equation (10). Apart from that, all studentized residuals fall within the range -3 < *r*_i_ < +3, indicating no outliers to CHI_IAM_ models. All these lead to the conclusion that the models based on pharmaceutical ecotoxicity can be well used to predict the aquatic toxicity of the UV filter compounds.

Similar conclusions can be drawn based on the log *P* values of the compounds. The largest log *P* value of the pharmaceutical compounds used as a training set is that of the drug amitryptiline (log *P* = 5.04) when five UV filters exhibit log *P* values even greater (octinoxate, octisalate, octocrylene, oxybenzone, padimate O). For example, octocrylene possesses a log *P* value equal to 7.27. However, all compounds fall again within the applicability domain of the models based on log *P* using the same criteria, as seen in Figures 1S and 2S in the Supplementary material.

#### Acute aquatic toxicity predictions - Comparison with log *P* and EPI Suite predictions

Since it was proven that the UV filters under investigation belong to the same chemical property space as that of pharmaceutical compounds, the previously established models for the prediction of pharmaceutical acute ecotoxicity based on CHI_IAM_ (Equations (1) and (3)) were used as well for UV filter acute ecotoxicity predictions. The same predictions were made based on previously established log *P* models (Equations (2) and (4)) for comparison.

Results for fish acute toxicity are shown in Table 4, where experimental, predicted by CHI_IAM_ (Equation (1)) and log *P* (Equation (2)) models and residual values of pLC_50_ are illustrated, along with corresponding predictions extracted from EPI Suite software. It should be noted that experimental and EPI predicted LC_50_ refer to 96 h static investigations, while CHI_IAM_ models were trained to predict 48 h LC_50_. However, this difference does not hinder the comparison of the values since fish LC_50_ values are not expected to vary considerably for short-term exposure testing [37-39].

**Table 4.** Experimental, predicted and residual values of fish *p*LC_50_

Compound	*p*LC_50_ fish				
	Experimental	Calculated	CHI_IAM_*	log P**	EPI
4-Aminobenzoic Acid	2.12	Predicted	2.56	2.45	2.12
		Residual	0.44	0.33	0.00
Ensulizole	2.91	Predicted	3.04	2.24	2.05
		Residual	0.13	0.67	0.86
Meradimate	4.48	Predicted	5.14	4.92	6.37
		Residual	0.66	0.44	1.89
Octisalate	4.40	Predicted	5.05	5.11	6.19
		Residual	0.65	0.71	1.79
Octocrylene	5.65	Predicted	5.29	6.02	6.73
		Residual	0.72	0.37	1.08
Oxybenzone	4.78	Predicted	4.36	4.14	4.92
		Residual	0.42	0.64	0.14
Padimate O	4.85	Predicted	4.98	5.20	6.07
		Residual	0.16	0.35	1.22
Sulisobenzone	2.69	Predicted	3.09	2.22	1.52
		Residual	0.40	0.47	1.17
Trolamine salicylate (Salicylic acid)	2.00	Predicted	2.46	1.69	3.26
		Residual	0.46	0.31	1.26

*Equation (1)

**Equation (2)

From the results of Table 4, it is evident that fish LC_50_ values closer to the experimental ones are predicted by the CHI_IAM_ model (Equation (1)) since these predictions possess the lowest residuals overall. Inferior predictions to those derived from the CHI_IAM_ model are followed by the model based on log *P* (Equation (2)), whereas the largest residuals and, consequently, the worst predictions occur using EPI Suite software. These differences are reflected also in the errors in the prediction. As shown in Table 5, the lowest prediction error parameters prioritized for the accuracy of the predictions (RMSEP and RSEP) belong to the predictions of the CHI_IAM_ model.

**Table 5.** Error parameters of the estimates of UV filter fish pLC_50_ values

	log *P*	CHI_IAM_	EPI
RMSEP	0.55	0.42	1.16
RSEP, %	13.8	10.4	28.9
bias	0.09	0.18	0.54

**Figure 6.**
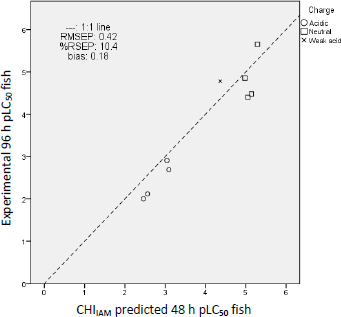
Experimental *vs.* CHI_IAM_ predicted fish pLC_50_ values

A graphical illustration of the prediction errors of the CHI_IAM_ model is shown in Figure 6 by plotting the experimental *vs.* the predicted values and, that way, depicting their distances from the 1:1 line. Figure 6 shows that uncharged compounds tend to be more toxic than negatively charged compounds since these are found in the upper section of the graph and the latter in the lower. The presence of a negative charge lowers the lipophilicity of the compounds (at physiological pH), thus rendering them less toxic. The repulsing electrostatic forces with the negative charge that predominates in the hydrophobic core of the phospholipids can explain the decreased toxic potential [20,29]. The same picture results by plotting the same graphs for log *P* model (Figure 3S) and EPI Suite (Figure 4S) predictions, which are included in the Supplemental data.

Similarly, Table 6 includes the experimental, predicted and residual values of *daphnia* pEC_50_ and pLC_50_, where the predicted values result from the CHI_IAM_ model (Equation (3)), log *P* model (Equation (4)) and EPI Suite software. All values refer to 48 h static investigations; EPI Suite, however, calculates only 48 h LC_50_ values and not EC_50_. Since experimental and model-predicted EC_50_ values express the sublethal effect of immobilization, LC_50_ and EC_50_ values are not considered far away regarding the quantitative information they express [40,41].

**Table 6.** Experimental, predicted and residual values of *daphnia* pEC_50_/pLC_50_

Compound	pEC_50_/pLC_50_ water flea (*Daphnia magna)*				
	Experimental	Calculated	CHI_IAM_*	log P**	EPI
4-Aminobenzoic Acid	3.26	Predicted	2.82	2.74	4.14
		Residual	0.44	0.52	0.88
Avobenzone	5.20	Predicted	4.72	4.53	5.64
		Residual	0.48	0.67	0.44
Dioxybenzone	4.76	Predicted	4.21	3.98	4.78
		Residual	0.55	0.78	0.02
Ensulizole	3.44	Predicted	3.19	2.58	3.04
		Residual	0.25	0.86	0.40
Meradimate	5.24	Predicted	4.86	4.62	6.25
		Residual	0.38	0.62	1.01
Octinoxate	5.03	Predicted	4.75	4.95	5.95
		Residual	0.28	0.08	0.92
Octisalate	5.58	Predicted	4.79	4.76	6.06
		Residual	0.79	0.82	0.48
Octocrylene	5.06	Predicted	4.97	5.43	6.63
		Residual	0.09	0.37	1.57
Oxybenzone	4.92	Predicted	4.24	4.23	5.15
		Residual	0.68	0.69	0.23
Padimate O	4.69	Predicted	4.73	4.82	5.93
		Residual	0.04	0.13	1.24
Sulisobenzone	3.79	Predicted	3.23	2.57	2.36
		Residual	0.56	1.22	1.43
Trolamine Salicylate (Salicylic acid)	2.67	Predicted	2.73	2.17	2.74
		Residual	0.06	0.50	0.07

*Equation (1)

**Equation (2)

By comparing the residual values of the predictions from Table 6, similar conclusions can be drawn for *daphnia* as for fish acute toxicity prediction. As in the case of fish, the best predictions for water fleas result from the application of the CHI_IAM_ model (Equation (3)), followed by predictions of the log *P* model (Equation (4)) and lastly by those of the EPI Suite. The prediction errors of Table 7 further support this conclusion since the predictions of the CHI_IAM_ model possess the lowest uncertainty, relative standard error and bias.

**Table 7.** Error parameters of the estimates of UV filter fish *p*EC_50_/*p*LC_50_ values

	log *P*	CHI_IAM_	EPI
RMSEP	0.65	0.45	0.88
RSEP, %	14.5	10.1	19.8
bias	-0.46	-0.17	0.42

**Figure 7.**
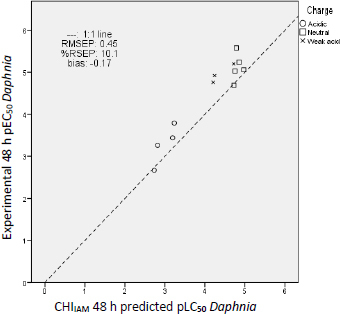
Experimental *vs.* CHI_IAM_ predicted *Daphnia* pEC_50_ values

The distances of the predictions from the 1:1 line are presented in Figure 7 for the CHI_IAM_ model, while the corresponding errors are illustrated in Figure 5S and Figure 6S for the log *P* model and EPI Suite predictions, respectively. As in the previous case of fish toxicity, charge plays an important role in the toxicity potential, with negative charge lowering the toxic impact of a compound. The same toxicity mechanism was scrutinized in our previous work on pharmaceutical ecotoxicity [20] and proves that UV filter compounds act the same way as pharmaceutical compounds regarding their acute aquatic toxicity. It should be noted that the compounds shown as weak acids in Figure 7 (avobenzone, dioxybenzone) have a negatively charged molar fraction of only 10 to 20 %. This fraction is insufficient to significantly reduce their phospholipid binding and, consequently, their potential toxicity.

From all the above, it is clear that CHI_IAM_ contains more information than log *P* and can predict the acute aquatic toxicity of UV filter compounds more accurately than octanol-water. CHI_IAM_ incorporates electrostatic interactions with compounds absent in log *P* measurements. In addition, CHI_IAM_ is a real-time chromatographic measurement of the interaction of a compound with phospholipids, thus avoiding predictions based on calculated values, such as calculated log *P* [42], which can lead to great over- or underestimations and uncertainty of the predictions. One such example is the predictions of EPI Suite that are based on calculated log *P*. EPI Suite is an *in silico* approach for the prediction of aquatic toxicity endpoints, and although it offers rapid predictions without the use of measurements, it contains limitations, as it lacks the precision that measured data can offer. As shown, EPI Suite led to much larger errors in the estimation of toxicity endpoints than CHI_IAM_. Regarding the experimental determination of log *P*, chromatographic measurements are faster, more user-friendly, and can be automated [43].

#### Prediction of ecotoxicological endpoints for UV filters with no literature data

Considering that phospholipid binding can successfully describe the acute toxic action of UV filters on aquatic organisms, the CHI_IAM_ models were used to predict ecotoxicological endpoints of compounds for which toxicity data could not be found in the literature. Predicted 48 h *p*LC_50_ values to fish are presented in Table 8, while predicted 48 h *p*EC_50_ values to water fleas *(Daphnia magna)* are presented in Table 9. The predicted values were then converted from their molar p values to mg/L to be classified based on the occurring values according to Global Harmonized System (GHS) aquatic hazard classification [44].

**Table 8.** Predicted fish 48 h LC_50_ of UV filters with no literature data

Compound	Predicted 48 h pLC_50_, M*	Predicted 48 h LC_50_, mg/L	Hazard ranking
Avobenzone	4.97	3.35	Acute toxicity II / Toxic
Dioxybenzone	4.32	11.7	Acute toxicity III / Harmful
Homosalate	5.13	1.93	Acute toxicity II / Toxic
Octinoxate	5.00	2.84	Acute toxicity II / Toxic

*Equation (1)

As seen from Tables 8 and 9, all compounds are classified as hazardous to aquatic organisms and, more specifically, as acutely toxic or harmful. This fact can be justified considering that these specific compounds belong to the more lipophilic group of compounds investigated. Taking into account that the UV filter effect is not related to the lipophilicity of compounds and that such compounds are actually widely used in cosmetic formulations and sunscreen products, ending up eventually in the aquatic environment, sun protection should be directed to the use of less lipophilic compounds that can absorb the harmful UV rays of light, in order to achieve more environmentally friendly sun protection products.

**Table 9.** Predicted daphnid (water flea, *Daphnia magna*) 48 h EC_50_ of UV filters with no literature data

Compound	Predicted 48 h pEC_50_, M*	Predicted 48 h EC_50_, mg/L	Hazard ranking
Homosalate	4.85	3.71	Acute toxicity II / Toxic

*Equation (3)

## Conclusions

UV filter compounds share similar chemical properties with pharmaceutical compounds, allowing models developed for predicting the acute aquatic toxicity of pharmaceuticals to be applied to UV filters. Phospholipid binding has proven to be a more successful descriptor of the acute toxic action of UV filters than the traditionally used octanol-water partition coefficient (log *P*). The CHI_IAM_ descriptor provides more comprehensive information than log *P*, capturing electrostatic interactions through chromatographic measurements of real interactions between compounds and phospholipids. Using this biomimetic descriptor for ecotoxicity predictions can overcome problems such as discrepancies, deviations, and uncertainties that arise from the calculated log *P* values used by most software programs (*e.g*., EPI Suite). The acute toxic action of UV filters is influenced primarily by lipophilicity, with charge playing a significant role; a negative charge reduces a compound's toxicity. Most investigated UV filters are uncharged and highly lipophilic, leading to strong phospholipid binding and, consequently, greater aquatic toxicity. These characteristics make the use of these compounds in cosmetic formulations hazardous for aquatic organisms, especially given the large quantities of sunscreen products used during the summer season. Skincare and sun protection should focus on using less lipophilic UV filters, preferably with a negative charge, to decrease phospholipid binding and create more environmentally friendly formulations and products.

## Supplementary material

Additional data are available at https://pub.iapchem.org/ojs/index.php/admet/article/view/2364, or from the corresponding author on request.


