# Supplementary material for: Predicting the acute aquatic toxicity of organic UV filters used in cosmetic formulations
Source: ADMET DMPK. 2024 Sep 11;12(5):781–96. doi: 10.5599/admet.2364 (PMC11542717; doi:10.5599/admet.2364)
Supplement: Supplementary file 1 [file ADMET-12-2364-S1.pdf]

ADMET & DMPK 12(5) (2024) S13-S17

Open Access : ISSN : 1848-7718

http://www.pub.iapchem.org/ojs/index.php/admet/index

Supplementary material to

Predicting the acute aquatic toxicity of organic UV filters used in cosmetic formulations

Chrysanthos Stergiopoulos[1,*], Fotios Tsopelas[1], Maria Ochsenkühn-Petropoulou[1] and Klara Valko[2]

[1]*Laboratory of Inorganic and Analytical Chemistry, School of Chemical Engineering, National Technical University of Athens, Iroon Polytechneiou 9, Zografou 157 80, Athens, Greece*
[2]*Business & Technology Centre, Bessemer Drive, Stevenage, Herts, SG1 2DX, United Kingdom*

ADMET & DMPK **12(5)** (2024) 781-796; https://doi.org/10.5599/admet.2364

Table 1S Experimental ecotoxicological values of compounds investigated

Compound	pLC_{50} 96 h, M - fish	pEC_{50} 48 h, M - water flea *(Daphnia magna)*
4-Aminobenzoic Acid	2.12	3.26
Avobenzone	n/d	5.20
Dioxybenzone	n/d	4.76
Ensulizole	2.91	3.44
Homosalate	n/d	n/d
Meradimate	4.48	5.24
Octinoxate	n/d	5.03
Octisalate	4.40	5.58
Octocrylene	5.65	5.06
Oxybenzone	4.78	4.92
Padimate O	4.85	4.69
Sulisobenzone	2.69	3.79
Trolamine Salicylate	2.00	2.67

LC_{50}: lethal concentration to 50 % of the population, EC_{50}: effective concentration of 50 % of the population (immobilization); n/d: no data

Table 2S CHI_{IAM} values of pharmaceuticals used for model construction (training set)

Pharmaceutical	CHI_{IAM}
Acetaminophen	2.40
Acetanilide	11.9
Amitriptyline	55.3
Ampicillin	10.5
Atenolol	15.8
Caffeine	3.18
Carbamazepine	26.9
Clofibrate	40.5
Diclofenac	34.1
Diltiazem	42.0
Fenofibrate	46.8
Fluvoxamine	50.2
Gemfibrozil	33.7
Ibuprofen	20.6
Metoprolol	35.4
Metronidazole	-4.81
Ofloxacin	28.9
Oxiconazole	53.7
Propranolol	39.5
Pyrimethamine	38.2
Salicylic acid	-8.67

Pharmaceutical	CHI_{IAM}
Sulfachlorpyridazine	7.67
Sulfadimethoxine	13.8
Sulfamethoxazole	-3.19
Sulfamethoxypyridazine	10.9
Sulfathiazole	11.0
Theophylline	1.80
Verapamil	44.8
Warfarin	23.5

CHI_{IAM}: chromatographic hydrophobicity index on immobilized artificial membrane column

Table 3S Physicochemical parameters of pharmaceuticals used for model construction (training set)

Compound	log *P*	log $D_{7.4}$	MW	TPSA	F^+	F^-	F^z	*A*	*B*	HBD	HBA
Acetaminophen	0.46	0.23	151	49.3	0.00	0.00	0.00	1.04	0.86	2.00	3.00
Acetanilide	1.16	1.05	135	29.1	0.00	0.00	0.00	0.48	0.67	1.00	2.00
Amitriptyline	5.04	3.70	277	3.24	0.98	0.00	0.00	0.00	1.00	0.00	1.00
Ampicillin	-1.33	-3.30	349	138.0	0.00	0.53	0.47	1.06	2.62	4.00	7.00
Atenolol	0.16	-1.89	266	84.6	0.99	0.00	0.00	0.69	2.00	4.00	5.00
Caffeine	-0.07	-0.45	194	58.4	0.00	0.00	0.00	0.05	1.28	0.00	6.00
Carbamazepine	2.30	2.58	236	46.3	0.00	0.00	0.00	0.53	1.10	2.00	3.00
Clofibrate	3.52	3.52	243	35.5	0.00	0.00	0.00	0.00	0.69	0.00	3.00
Diclofenac	4.40	1.23	296	49.3	0.00	1.00	0.00	0.63	0.96	2.00	3.00
Diltiazem	2.70	1.97	415	84.4	0.88	0.00	0.00	0.00	2.12	0.00	6.00
Fenofibrate	4.38	4.38	361	52.6	0.00	0.00	0.00	0.00	1.13	0.00	4.00
Fluvoxamine	3.63	2.32	318	56.8	0.96	0.00	0.00	0.23	1.14	2.00	4.00
Gemfibrozil	4.39	1.90	250	46.5	0.00	1.00	0.00	0.57	0.71	1.00	3.00
Ibuprofen	3.50	0.38	206	37.3	0.00	1.00	0.00	0.59	0.81	1.00	2.00
Metoprolol	1.88	-0.43	267	50.7	0.99	0.00	0.00	0.17	1.76	2.00	4.00
Metronidazole	-0.27	-0.27	171	86.9	0.00	0.00	0.00	0.18	1.03	1.00	6.00
Ofloxacin	-0.39	-2.34	361	73.3	0.05	0.10	0.85	0.57	2.05	1.00	7.00
Oxiconazole	4.89	4.53	429	39.4	0.58	0.00	0.00	0.00	0.91	0.00	4.00
Propranolol	3.09	0.89	259	41.5	0.99	0.00	0.00	0.17	1.42	2.00	3.00
Pyrimethamine	2.69	2.16	249	77.8	0.58	0.00	0.00	0.45	0.99	4.00	4.00
Salicylic acid	2.26	-1.89	138	57.5	0.00	1.00	0.00	0.71	0.38	2.00	3.00
Sulfachlorpyridazine	0.79	0.18	325	106.4	0.00	0.74	0.00	0.64	1.38	3.00	6.00
Sulfadimethoxine	1.63	0.02	310	124.8	0.00	0.96	0.00	0.59	1.78	3.00	8.00
Sulfamethoxazole	0.32	-0.85	253	106.6	0.00	0.98	0.00	0.59	1.21	3.00	6.00
Sulfamethoxypyridazine	0.28	-0.01	280	115.6	0.00	0.74	0.00	0.59	1.41	3.00	7.00
Sulfathiazole	0.87	0.62	255	121.7	0.00	0.42	0.00	0.59	1.21	3.00	5.00
Theophylline	0.12	0.10	180	69.3	0.00	0.04	0.00	0.54	1.34	1.00	6.00
Verapamil	3.83	3.17	455	63.9	0.98	0.00	0.00	0.00	1.89	0.00	6.00
Warfarin	2.60	-0.26	308	63.6	0.00	1.00	0.00	0.35	1.49	1.00	4.00

P: partition coefficient, $D_{7.4}$: distribution coefficient at pH 7.4, MW: molecular weight, F^+: positively charged molecular fraction, F^-: negatively charged molecular fraction, F^z: zwitterionically charged molecular fraction, *A*: Abraham's acidity parameter, *B*: Abraham's basicity parameter, HBD: hydrogen bond donors, HBA: hydrogen bond acceptors

Table 4S Calibration set of compounds used and their predetermined CHI_{IAM} values

Compound	CHI_{IAM}
Acetanilide	11.5
Acetophenone	17.2
Butyrophenone	32.0
Heptanophenone	45.7
Hexanophenone	41.8
Octanophenone	49.4
Paracetamol	2.91
Propiophenone	25.9
Valerophenone	37.3

CHI_{IAM}: chromatographic hydrophobicity index on immobilized artificial membrane column

(cc) BY

Table 5S EPI Suite predicted ecotoxicity values of UV-filter compounds

Compounds	pLC_{50} 96 h, M - fish	pLC_{50} 48 h, M - water flea *(Daphnia magna)*
4-Aminobenzoic Acid	2.12	4.14
Avobenzone	5.69	5.64
Dioxybenzone	5.36	4.78
Ensulizole	2.05	3.04
Homosalate	6.30	6.18
Meradimate	6.37	6.25
Octinoxate	6.09	5.95
Octisalate	6.19	6.06
Octocrylene	6.73	6.63
Oxybenzone	4.92	5.15
Padimate O	6.07	5.93
Sulisobenzone	1.52	2.36
Trolamine Salicylate	3.26	2.74

LC_{50}: lethal concentration to 50 % of the population

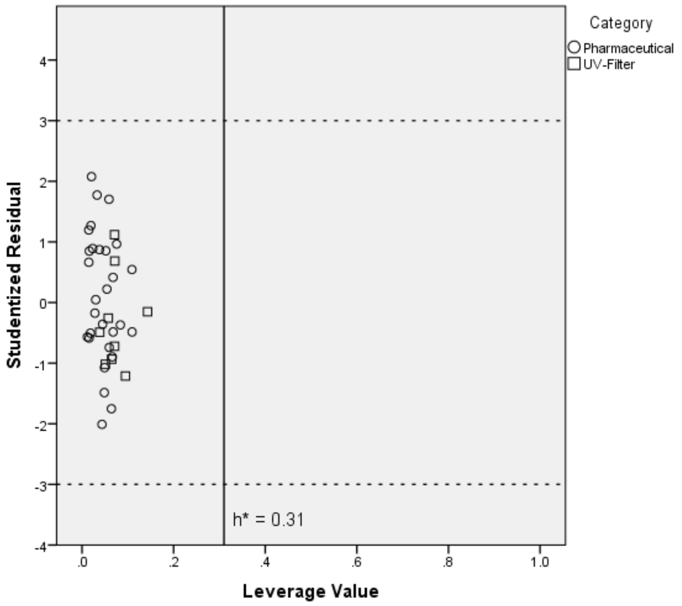

Figure 1S Applicability domain of fish pLC_{50} logP predictive model

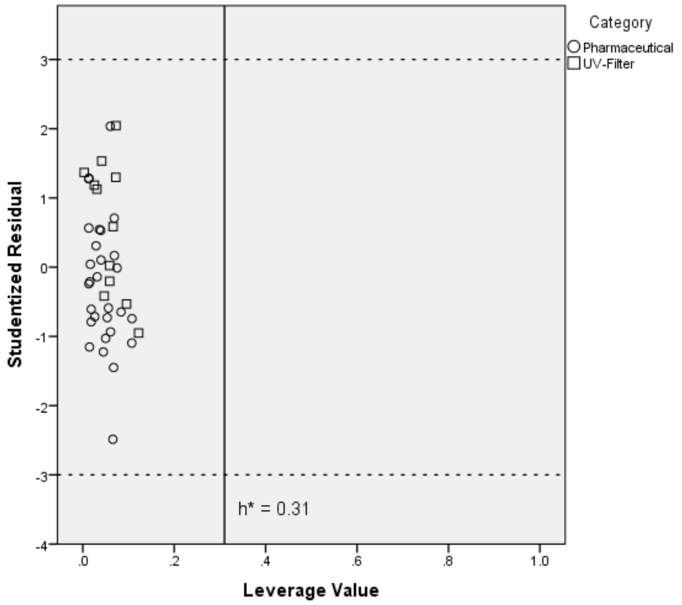

Figure 2S Applicability domain of daphnia pEC_{50} logP predictive model

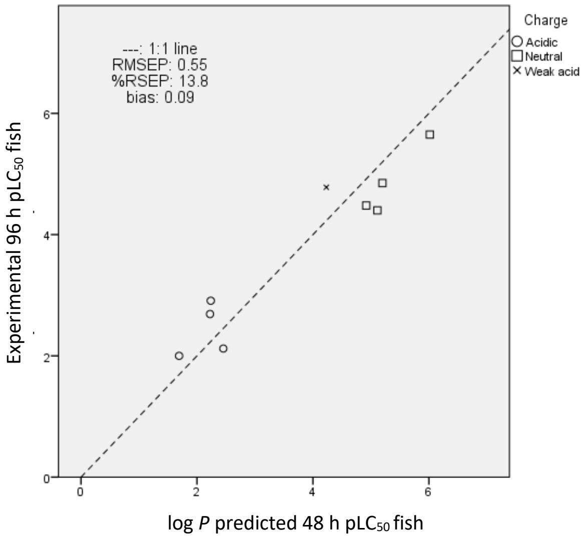

Figure 3S Experimental *vs.* log *P* predicted fish pLC_{50} values

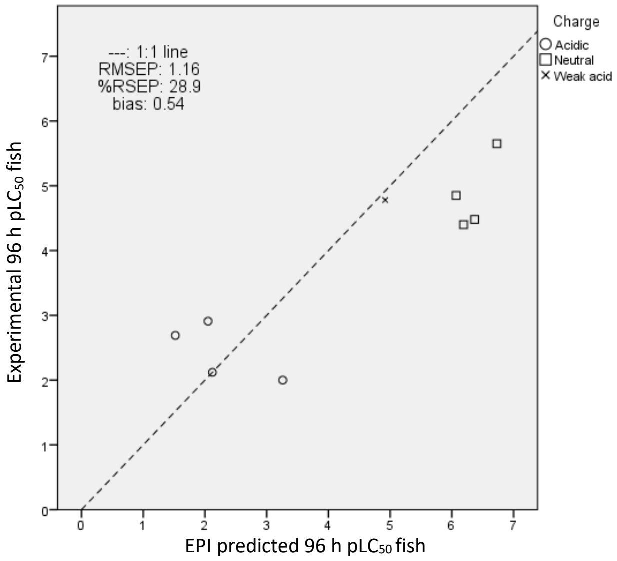

Figure 4S Experimental v*s.* EPI predicted fish pLC_{50} values

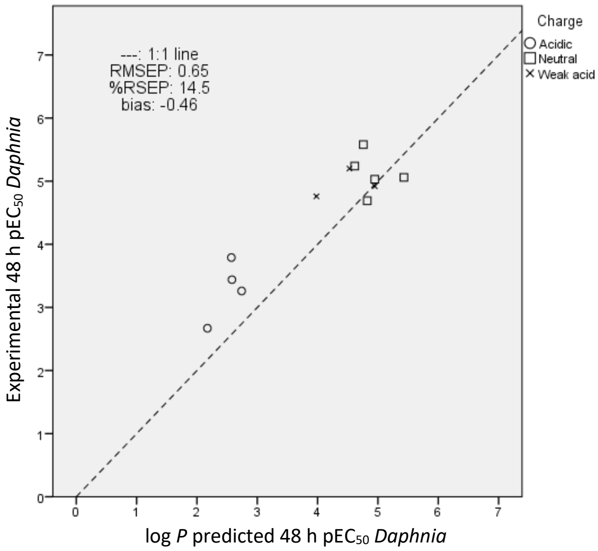

Figure 5S Experimental *vs.* log *P* predicted *Daphnia* pEC_{50} values

(cc) BY

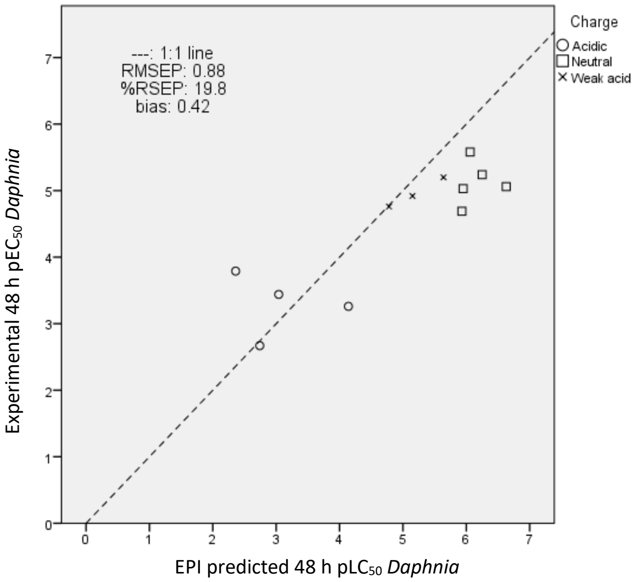

Figure 6S Experimental *vs.* EPI predicted daphnia pEC_{50} / pLC_{50} values
