# Supplementary material for: Predicting the acute aquatic toxicity of organic UV filters used in cosmetic formulations
Source: ADMET DMPK. 2024 Sep 11;12(5):781–96. doi: 10.5599/admet.2364 (PMC11542717; doi:10.5599/admet.2364)
Supplement: Supplementary file 2 [file ADMET-12-2364-S1.docx]

*ADMET & DMPK 12(5) (2024) S13-S17*

*
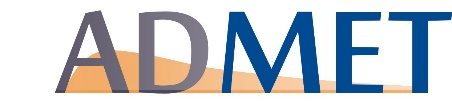
***Open Access : ISSN : 1848-7718**[***http://www.pub.iapchem.org/ojs/index.php/admet/index***](http://www.pub.iapchem.org/ojs/index.php/admet/index)

Supplementary material to

**Predicting the acute aquatic toxicity of organic UV filters used in cosmetic formulations**

Chrysanthos Stergiopoulos^1,^*, Fotios Tsopelas^1^, Maria Ochsenkühn-Petropoulou^1^ and Klara Valko^2^

*^1^Laboratory of Inorganic and Analytical Chemistry, School of Chemical Engineering, National Technical University of Athens, Iroon Polytechneiou 9, Zografou 157 80, Athens, Greece*

*^2^Business & Technology Centre, Bessemer Drive, Stevenage, Herts, SG1 2DX, United Kingdom*

ADMET & DMPK **12(5)** (2024) 781-796; <https://doi.org/10.5599/admet.2364>

**Table 1S** Experimental ecotoxicological values of compounds investigated

| Compound | pLC_50_ 96 h, M - fish | pEC_50_ 48 h, M - water flea *(Daphnia magna)* |
| --- | --- | --- |
| 4-Aminobenzoic Acid | 2.12 | 3.26 |
| Avobenzone | n/d | 5.20 |
| Dioxybenzone | n/d | 4.76 |
| Ensulizole | 2.91 | 3.44 |
| Homosalate | n/d | n/d |
| Meradimate | 4.48 | 5.24 |
| Octinoxate | n/d | 5.03 |
| Octisalate | 4.40 | 5.58 |
| Octocrylene | 5.65 | 5.06 |
| Oxybenzone | 4.78 | 4.92 |
| Padimate O | 4.85 | 4.69 |
| Sulisobenzone | 2.69 | 3.79 |
| Trolamine Salicylate | 2.00 | 2.67 |

LC_50_: lethal concentration to 50 % of the population, EC_50_: effective concentration of 50 % of the population (immobilization); n/d: no data

**Table 2S** CHI_IAM_ values of pharmaceuticals used for model construction (training set)

| Pharmaceutical | CHI_IAM_ |
| --- | --- |
| Acetaminophen | 2.40 |
| Acetanilide | 11.9 |
| Amitriptyline | 55.3 |
| Ampicillin | 10.5 |
| Atenolol | 15.8 |
| Caffeine | 3.18 |
| Carbamazepine | 26.9 |
| Clofibrate | 40.5 |
| Diclofenac | 34.1 |
| Diltiazem | 42.0 |
| Fenofibrate | 46.8 |
| Fluvoxamine | 50.2 |
| Gemfibrozil | 33.7 |
| Ibuprofen | 20.6 |
| Metoprolol | 35.4 |
| Metronidazole | -4.81 |
| Ofloxacin | 28.9 |
| Oxiconazole | 53.7 |
| Propranolol | 39.5 |
| Pyrimethamine | 38.2 |
| Salicylic acid | -8.67 |
| Sulfachlorpyridazine | 7.67 |
| Sulfadimethoxine | 13.8 |
| Sulfamethoxazole | -3.19 |
| Sulfamethoxypyridazine | 10.9 |
| Sulfathiazole | 11.0 |
| Theophylline | 1.80 |
| Verapamil | 44.8 |
| Warfarin | 23.5 |

CHI_IAM_: chromatographic hydrophobicity index on immobilized artificial membrane column

**Table 3S** Physicochemical parameters of pharmaceuticals used for model construction (training set)

| Compound | log *P* | log *D*_7.4_ | MW | TPSA | *F*^+^ | *F*^-^ | *F*^z^ | *A* | *B* | HBD | HBA |
| --- | --- | --- | --- | --- | --- | --- | --- | --- | --- | --- | --- |
| Acetaminophen | 0.46 | 0.23 | 151 | 49.3 | 0.00 | 0.00 | 0.00 | 1.04 | 0.86 | 2.00 | 3.00 |
| Acetanilide | 1.16 | 1.05 | 135 | 29.1 | 0.00 | 0.00 | 0.00 | 0.48 | 0.67 | 1.00 | 2.00 |
| Amitriptyline | 5.04 | 3.70 | 277 | 3.24 | 0.98 | 0.00 | 0.00 | 0.00 | 1.00 | 0.00 | 1.00 |
| Ampicillin | -1.33 | -3.30 | 349 | 138.0 | 0.00 | 0.53 | 0.47 | 1.06 | 2.62 | 4.00 | 7.00 |
| Atenolol | 0.16 | -1.89 | 266 | 84.6 | 0.99 | 0.00 | 0.00 | 0.69 | 2.00 | 4.00 | 5.00 |
| Caffeine | -0.07 | -0.45 | 194 | 58.4 | 0.00 | 0.00 | 0.00 | 0.05 | 1.28 | 0.00 | 6.00 |
| Carbamazepine | 2.30 | 2.58 | 236 | 46.3 | 0.00 | 0.00 | 0.00 | 0.53 | 1.10 | 2.00 | 3.00 |
| Clofibrate | 3.52 | 3.52 | 243 | 35.5 | 0.00 | 0.00 | 0.00 | 0.00 | 0.69 | 0.00 | 3.00 |
| Diclofenac | 4.40 | 1.23 | 296 | 49.3 | 0.00 | 1.00 | 0.00 | 0.63 | 0.96 | 2.00 | 3.00 |
| Diltiazem | 2.70 | 1.97 | 415 | 84.4 | 0.88 | 0.00 | 0.00 | 0.00 | 2.12 | 0.00 | 6.00 |
| Fenofibrate | 4.38 | 4.38 | 361 | 52.6 | 0.00 | 0.00 | 0.00 | 0.00 | 1.13 | 0.00 | 4.00 |
| Fluvoxamine | 3.63 | 2.32 | 318 | 56.8 | 0.96 | 0.00 | 0.00 | 0.23 | 1.14 | 2.00 | 4.00 |
| Gemfibrozil | 4.39 | 1.90 | 250 | 46.5 | 0.00 | 1.00 | 0.00 | 0.57 | 0.71 | 1.00 | 3.00 |
| Ibuprofen | 3.50 | 0.38 | 206 | 37.3 | 0.00 | 1.00 | 0.00 | 0.59 | 0.81 | 1.00 | 2.00 |
| Metoprolol | 1.88 | -0.43 | 267 | 50.7 | 0.99 | 0.00 | 0.00 | 0.17 | 1.76 | 2.00 | 4.00 |
| Metronidazole | -0.27 | -0.27 | 171 | 86.9 | 0.00 | 0.00 | 0.00 | 0.18 | 1.03 | 1.00 | 6.00 |
| Ofloxacin | -0.39 | -2.34 | 361 | 73.3 | 0.05 | 0.10 | 0.85 | 0.57 | 2.05 | 1.00 | 7.00 |
| Oxiconazole | 4.89 | 4.53 | 429 | 39.4 | 0.58 | 0.00 | 0.00 | 0.00 | 0.91 | 0.00 | 4.00 |
| Propranolol | 3.09 | 0.89 | 259 | 41.5 | 0.99 | 0.00 | 0.00 | 0.17 | 1.42 | 2.00 | 3.00 |
| Pyrimethamine | 2.69 | 2.16 | 249 | 77.8 | 0.58 | 0.00 | 0.00 | 0.45 | 0.99 | 4.00 | 4.00 |
| Salicylic acid | 2.26 | -1.89 | 138 | 57.5 | 0.00 | 1.00 | 0.00 | 0.71 | 0.38 | 2.00 | 3.00 |
| Sulfachlorpyridazine | 0.79 | 0.18 | 325 | 106.4 | 0.00 | 0.74 | 0.00 | 0.64 | 1.38 | 3.00 | 6.00 |
| Sulfadimethoxine | 1.63 | 0.02 | 310 | 124.8 | 0.00 | 0.96 | 0.00 | 0.59 | 1.78 | 3.00 | 8.00 |
| Sulfamethoxazole | 0.32 | -0.85 | 253 | 106.6 | 0.00 | 0.98 | 0.00 | 0.59 | 1.21 | 3.00 | 6.00 |
| Sulfamethoxypyridazine | 0.28 | -0.01 | 280 | 115.6 | 0.00 | 0.74 | 0.00 | 0.59 | 1.41 | 3.00 | 7.00 |
| Sulfathiazole | 0.87 | 0.62 | 255 | 121.7 | 0.00 | 0.42 | 0.00 | 0.59 | 1.21 | 3.00 | 5.00 |
| Theophylline | 0.12 | 0.10 | 180 | 69.3 | 0.00 | 0.04 | 0.00 | 0.54 | 1.34 | 1.00 | 6.00 |
| Verapamil | 3.83 | 3.17 | 455 | 63.9 | 0.98 | 0.00 | 0.00 | 0.00 | 1.89 | 0.00 | 6.00 |
| Warfarin | 2.60 | -0.26 | 308 | 63.6 | 0.00 | 1.00 | 0.00 | 0.35 | 1.49 | 1.00 | 4.00 |

*P*: partition coefficient, *D*_7.4_: distribution coefficient at pH 7.4, MW: molecular weight, *F*^+^: positively charged molecular fraction, *F*^-^: negatively charged molecular fraction, *F*^z^: zwitterionically charged molecular fraction, *A*: Abraham’s acidity parameter, *B*: Abraham’s basicity parameter, HBD: hydrogen bond donors, HBA: hydrogen bond acceptors

**Table 4S** Calibration set of compounds used and their predetermined CHI_IAM_ values

| Compound | CHI_IAM_ |
| --- | --- |
| Acetanilide | 11.5 |
| Acetophenone | 17.2 |
| Butyrophenone | 32.0 |
| Heptanophenone | 45.7 |
| Hexanophenone | 41.8 |
| Octanophenone | 49.4 |
| Paracetamol | 2.91 |
| Propiophenone | 25.9 |
| Valerophenone | 37.3 |

CHI_IAM_: chromatographic hydrophobicity index on immobilized artificial membrane column

**Table 5S** EPI Suite predicted ecotoxicity values of UV-filter compounds

| Compounds | pLC_50_ 96 h, M - fish | pLC_50_ 48 h, M - water flea *(Daphnia magna)* |
| --- | --- | --- |
| 4-Aminobenzoic Acid | 2.12 | 4.14 |
| Avobenzone | 5.69 | 5.64 |
| Dioxybenzone | 5.36 | 4.78 |
| Ensulizole | 2.05 | 3.04 |
| Homosalate | 6.30 | 6.18 |
| Meradimate | 6.37 | 6.25 |
| Octinoxate | 6.09 | 5.95 |
| Octisalate | 6.19 | 6.06 |
| Octocrylene | 6.73 | 6.63 |
| Oxybenzone | 4.92 | 5.15 |
| Padimate O | 6.07 | 5.93 |
| Sulisobenzone | 1.52 | 2.36 |
| Trolamine Salicylate | 3.26 | 2.74 |

LC_50_: lethal concentration to 50 % of the population


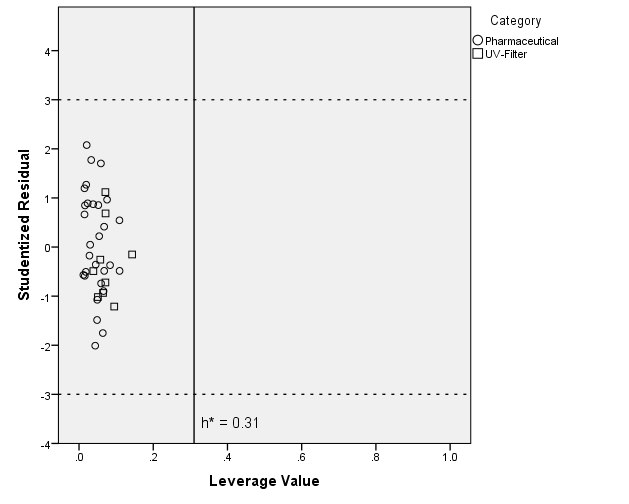


**Figure 1S** Applicability domain of fish pLC_50_ logP predictive model


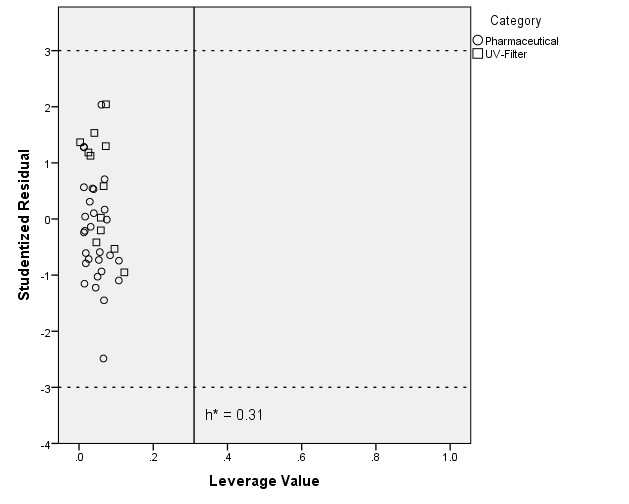


**Figure 2S** Applicability domain of daphnia pEC_50_ logP predictive model


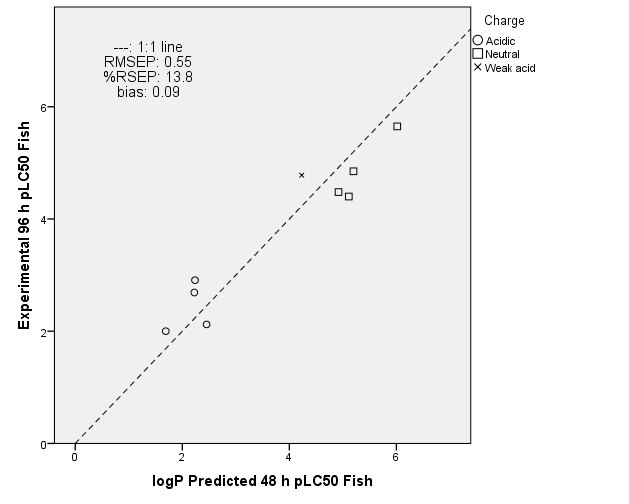


Experimental 96 h pLC_50_ fish

log *P* predicted 48 h pLC_50_ fish

**Figure 3S** Experimental *vs.* log *P* predicted fish pLC_50_ values


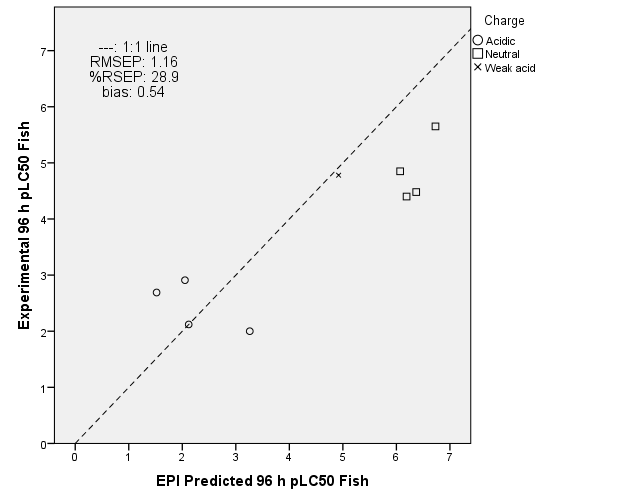


Experimental 96 h pLC_50_ fish

EPI predicted 96 h pLC_50_ fish

**Figure 4S** Experimental vs. EPI predicted fish pLC_50_ values


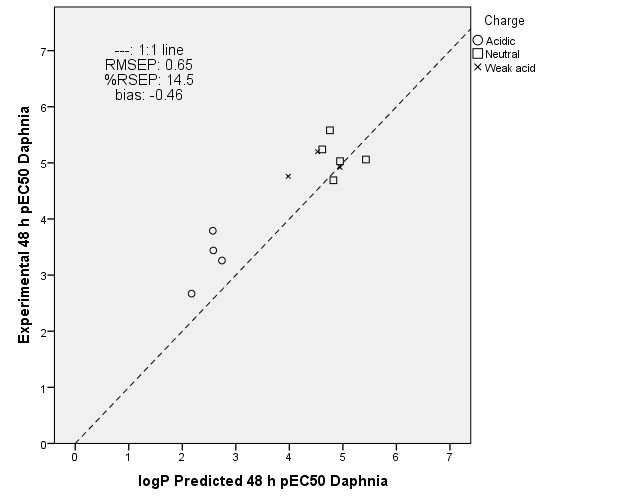


Experimental 48 h pEC_50_ *Daphnia*

log *P* predicted 48 h pEC_50_ *Daphnia*

**Figure 5S** Experimental vs. log P predicted Daphnia pEC_50_ values


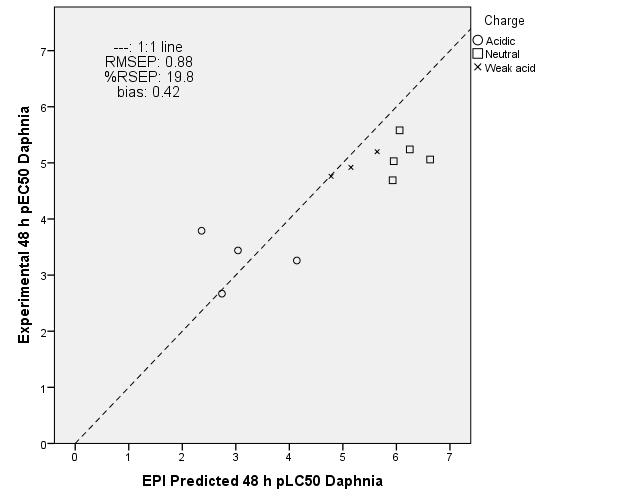


Experimental 48 h pEC_50_ *Daphnia*

EPI predicted 48 h pLC_50_ *Daphnia*

**Figure 6S** Experimental vs. EPI predicted daphnia pEC_50_ / pLC_50_ values
